# Effect of Dietary Exogenous Enzyme Supplementation on Enteric Mucosal Morphological Development and Adherent Mucin Thickness in Turkeys

**DOI:** 10.3389/fvets.2015.00045

**Published:** 2015-10-13

**Authors:** Ayuub A. Ayoola, Ramon D. Malheiros, Jesse L. Grimes, Peter R. Ferket

**Affiliations:** ^1^Prestage Department of Poultry Science, North Carolina State University, Raleigh, NC, USA

**Keywords:** supplemental enzymes, gut health, enteric mucosal morphology, mucin, turkeys

## Abstract

Anti-nutritional factors (ANFs) in feed ingredients can challenge gut health and reduce nutrient utilization. Birds typically activate their innate immune system as a protective response against the adverse effects of ANF, which often involves the secretion of mucin. Although dietary supplementation of exogenous enzymes are commonly used to alleviate the adverse effects of ANF on apparent nutrient digestibility, little is known about how they affect gut health, particularly in relation to the morphological development and mucin secretion of enteric mucosa. We carried out two trials to examine the effect of dietary supplementation of different types of exogenous enzymes on gut health of by accessing the effect of jejunum morphological development and ileal enteric adherent mucin thickness layer in turkeys. Dietary β-mannanase supplementation reduced ileal adherent mucin thickness layer (804 vs 823 μg/g; *p* < 0.05), while a commercial blend of xylanase, amylase, and protease (XAP) reduced ileal adherent mucin layer thickness (589 vs 740 μg/g; *p* < 0.05); thus reducing the apparent endogenous loss of nutrients. Both enzyme supplements also affected gut morphological characteristics. In comparison to the control treatment, dietary β-mannanase supplementation improved the jejunum tip width (219 vs 161; *p* < 0.05), base width (367 vs 300; *p* < 0.05), surface area (509,870 vs 380, 157; *p* < 0.05) and villi height/crypt depth ratio (7.49 vs 5.70; *p* < 0.05), and XAP improved the crypt depth (*p* < 0.05). In conclusion, dietary supplementation of exogenous enzymes may help alleviate the adverse effects of ANF on nutrient utilization by directly or indirectly removing the mucosal irritation that stimulates enteric mucin secretion.

## Introduction

The gut is a very complex and diverse ecosystem, and maintenance of gut health is a high nutrient-consuming task. Croom et al. (1) reported that energy required for gut maintenance accounts for about 25% of the total basal metabolic needs of an animal. The requirements may even be higher during an event of enteric distress or microbial pathogen challenge, which can significantly impact the partitioning of energy and other nutrients away from growth, thus reducing the overall feed conversion efficiency. Gut health can be maintained by a balance between the protective function of intestinal mucosa mucin secretion and the symbiotic community of microorganisms that competitively exclude pathogenic microbes (2).

Dietary composition plays an important role in maintaining the healthy gut ecosystem. Non-starch polysaccharides (NSPs), such as arabinoxylan and β-mannan, are one of the dietary components that influence the gut ecosystem. Their complex water-soluble structure increases digesta viscosity (3), which may entrap macronutrients, such as fat, protein, and starch, and reduces digestive enzyme–substrate interactions (4). Consequently, digestion and nutrient absorption in the foregut is impeded, which adversely effects nutrient supply for growth and reduces feed conversion efficiency. Furthermore, viscous β-mannan and arabinoxlyan may carry undigested nutrients from foregut into the hindgut where they become substrate for the fermentation of competitive microflora that alters the enteric ecosystem stability (5).

Excessive NSPs in the diet, such as arabinoxylan and β-mannan, may lead to the proliferation of undesirable pathogenic intestinal microflora, such as *E. coli* and *Clostridium spp*. (5–7). These enteric pathogens initiate a mucosal inflammatory response, leading to enteric distress, and suppressed gut morphological development. Consequently, this could have a biological effect on intestinal health by changing the mucosal morphological architecture and diverting more nutrients away from productive purposes toward intestinal maintenance. Enteric inflammation, due to enteric pathogen challenge, is associated with the activation of the innate immune system, which is positively associated with the stimulation of intestinal mucous secretion (8). Mucin, which is secreted by the goblet cells, is the protein-rich component of mucous. Although, little information is available on the influence of dietary arabinoxylan and β-mannan on enteric mucosa morphological development and health, atrophic shortening and thickening of jejunum villi, along with increased number of goblet cells per villus, has been observed in broilers fed a diet containing β-glucans which is a similar NSPs (9).

To our knowledge, there are limited published data available on the influence of enzymes on intestinal health, particularly as it affects gut morphology and intestinal adherent mucin secretion in turkey poults. This paper is a report of results from two experiments that evaluated the influence of dietary enzyme supplementation of turkeys fed high and low-energy diets, modified by dietary fat supplementation, on gut health as assessed by changes in mucosa morphometric characteristics and adherent ileal mucosa mucin layer thickness.

## Materials and Methods

### Experiment 1

#### Experimental diet

The experiment was designed as a 2 × 2 factorial arrangement of two dietary inclusion levels of β-mannanase (0 and 0.05% CTCzyme^®^, CTCBio, Inc., Korea), supplying 0 and ~500 U endo-beta-d-mannanase/kg of diet, respectively, and two dietary levels of metabolizable energy (high and low) that differed by 150 kcal ME/kg by the addition of supplemental beef tallow fat. All experimental diets were corn-SBM based formulations. The high-energy basal diet was made and divided into two lots, and each was mixed with either 0.05% of vermiculite, or 0.05% of β-mannanase. A second low-energy basal diet (150 kcal/kg less than the high-energy basal diet) was also made and divided into two parts, each was also mixed with either 0.05% of vermiculite or 0.05% of β-mannanase. Vermiculite was added as a non-nutritional filler in place of the enzyme in the experimental control diets. All experimental diets were formulated to meet or exceed NRC (10) requirements for turkeys (Table 1). All diets were supplemented with 2.0% Celite^®^ (Celite Corp., Lompar, CA, USA) which served as an acid insoluble ash indigestible reference marker for the determination of digestibility coefficients. The calculated and analyzed composition of the experimental diets, including the supplemental enzymes, is reported in Table 1. All diets were produced at the North Carolina State University Feed Mill Educational Unit (Raleigh, NC, USA).

**Table 1:** **Dietary ingredient composition and nutrient composition of starter diets fed to turkey hens from 1 to 28 days of age**.

Ingredients	High energy		Low energy	

	% Dietary **β**-mannanase supplementation			

	0	0.05	0	0.05

	(% of Diet)			
Corn	30.56	30.56	34.98	34.98
Soybean meal	44.84	44.84	44.24	44.24
Soy hulls	6.11	6.11	6.03	6.03
Poultry meal	5.00	5.00	5.00	5.00
Fat beef tallow	5.82	5.82	2.09	2.09
Dical phosphate	3.03	3.03	3.03	3.03
Limestone	0.95	0.95	0.95	0.95
dl-Methionine	0.44	0.44	0.43	0.43
Sodium chloride	0.32	0.32	0.32	0.32
l-Lysine-HCl	0.26	0.26	0.27	0.27
Choline chloride	0.22	0.22	0.22	0.22
Trace mineral^a^	0.20	0.20	0.20	0.20
Vitamin premix^b^	0.15	0.15	0.15	0.15
Selenite premix^c^	0.05	0.05	0.05	0.05
Celite™	2.00	2.00	2.00	2.00
CTCzyme^d^	0.00	0.05	0.00	0.05
Filler (vermiculite)	0.05	0.00	0.05	0.00
**Calculated chemical composition**				
Dry matter (%)	93.34	93.34	92.72	92.72
ME poultry (kcal/kg)	2850	2850	2700	2700
Crude protein (%)	30.06	30.06	30.19	30.19
Crude fat (%)	8.04	8.04	4.92	4.92
Crude fiber (%)	4.34	4.34	4.37	4.37
Calcium (%)	4.34	4.34	4.37	4.37
Total phosphorus (%)	1.40	1.40	1.40	1.40
Avail. phosphorus poultry (%)	1.09	1.09	1.09	1.09

*^a^Each kilogram of mineral premix (0.1% inclusion) supplied the following per kg of complete feed: 60 mg Zn as ZnSO⋅HO; 60 mg Mn as MnSO⋅HO; 40 mg Fe as FeSO⋅HO; 5 mg Cu as CuSO; 1.25 mg I as Ca(IO); and 1 mg Co as CoSO*.

*^b^Each kilogram of vitamin premix (0.1% inclusion) supplied the following per kg of complete feed: vitamin A, 13,200 IU; cholecalciferol, 4000 IU; alpha-tocopherol, 66 IU; niacin, 110 mg; pantothenic acid, 22 mg; riboflavin, 13.2 mg; pyridoxine, 8 mg; menadione, 4 mg; folic acid, 2.2 mg; thiamin, 4 mg; biotin, 0.253 mg; vitamin B, 0.04 mg; ethoxyquin, 100 mg*.

*^c^NaSeO premix provided 0.3 mg Se/kg of complete feed*.

*^d^Enzyme provided 0.05% of beta-mannanase in diet*.

#### Bird Husbandry and Tissue Sampling

Four hundred thirty-two Nicholas hen poults were obtained from a commercial hatchery (Prestage Farms Hatchery, Clinton, NC, USA) and randomly assigned to one of 48 wire-floored cages with nine poults per cage (Alternative Design cages, Alternative Design Manufacturing and Supply, Inc., Siloam Springs, AR, USA). Each bird was identified with a numbered neck-tag in sequence for each replicate cage group. Four experimental treatment groups were randomly assigned among 48 cages. Feed and water was available *ad libitum*. Birds were reared until 28 days of age. From 1 to 7 days, the birds were provided 23 h of light to 1 h darkness, and 14 L:10 D after 7 days. On 7, 14, and 21 days, a 3-cm segment of the jejunum, from the end of the duodenal loop toward the Meckel’s diverticulum, was sampled from each of four poults per cage, gently flushed with saline, and fixed in 10% formalin solution for subsequent histological analysis. Another gut segment, representing 1 cm into the ileal section from the Meckel’s diverticulum, was collected from each of the same four poults per cage for mucin histochemical analysis.

### Experiment 2

#### Experimental Diets

The objective of the second experiment was to determine the effect of the dietary supplementation of an experimental blend of xylanase, amylase, and protease (XAP) and a direct-fed microbial (DFM) product (DuPont, St. Louis, MO, USA) on gut morphological development and ileal adherent mucin layer thickness in turkeys. The experiment was designed as a 2 × 4 factorial, with two inclusion levels of DDGS and four levels of different type of feed supplements [negative control (NC), 2% supplemental fat, XAP, and XAP + DFM]. Two experimental corn-soybean meal basal diets, containing 6 or 18% DDGS, were pelleted and crumbled and subsequently divided into four lots. The first lot was retained as the NC, the second lot was supplemented with 2% supplemental fat, the third lot was supplemented with the enzyme blend XAP, and the fourth lot was supplemented with the combination of XAP + DFM. The enzyme activities per gram of the XAP blend was 2000 FTU of xylanase, 200 FTU of amylase, and 5000 FTU of protease, and the XAP + DFM also contained 75,000 cfu of *Bacillus subtilis* per gram of diet. This same experimental diet preparation procedure was used for all feed phases. All experimental diets were formulated to meet or exceed the NRC (10) nutritional recommendations for turkeys (Table 2). All diets were manufactured at the North Carolina State University Feed Mill Educational Unit (Raleigh, NC, USA).

**Table 2:** **Dietary ingredients and nutrients composition fed to turkey hens in experiment 2**.

Ingredients	Starter		Grower 1		Grower 2	
	6% DDGS	18% DDGS	6% DDGS	18% DDGS	6% DDGS	18% DDGS

	(% of Diet)					
Soybean meal	42.65	44.37	35.59	31.07	31.99	26.65
Corn	39.25	28.74	46.43	38.83	50.44	43.74
DDGS	6.00	18.00	6.00	18.00	6.00	18.00
Poultry meal (60% CP)	5.00	3.00	5.00	5.00	5.00	5.00
Poultry fat	1.53	2.85	2.17	2.40	2.32	2.45
Dical P 18.5	2.39	2.60	1.92	1.64	1.51	1.25
Limestone	1.23	1.48	1.28	1.46	1.24	1.42
Alimet	0.55	0.53	0.41	0.37	0.38	0.35
l-Lysine HCl	0.35	0.39	0.29	0.37	0.22	0.33
Micro salt	0.32	0.33	0.30	0.26	0.27	0.24
Trace mineral premix^a^	0.20	0.20	0.20	0.20	0.20	0.20
Choline chloride 60%	0.19	0.16	0.19	0.14	0.18	0.14
Vitamin premix^b^	0.15	0.15	0.15	0.15	0.15	0.15
l-Threonine	0.11	0.13	0.02	0.02	0.02	0.02
Sodium selenite premix^c^	0.05	0.05	0.05	0.05	0.05	0.05
Feed supplement/filler^d^	0.02	0.02	0.02	0.02	0.02	0.02
Phytase	0.01	0.01	0.01	0.01	0.01	0.01
**Chemical analysis**						
Dry matter* (%)	88.3	89.97	90.35	90.5	90.3	89.38
ME poultry (kcal/kg)	2900	2900	3000	3000	3050	3050
Crude protein* (%)	29.6	29.1	26.01	26.49	24.4	23.5
Crude fat* (%)	5.91	6.18	6.63	7.25	6.13	7.7
Crude fiber (%)	2.68	3.33	2.55	3.04	2.49	2.97
Arabinoxylan (%)	1.80	2.75	2.02	3.04	2.12	3.18
Calcium* (%)	1.16	0.8	1.18	1.33	1.13	1.16
Total phosphorus* (%)	0.79	10.98	0.74	0.74	0.76	0.72
Avail. phosphorus poultry	0.8	0.8	0.7	0.7	0.62	0.62
Lysine (%)	1.889	1.894	1.648	1.669	1.4917	1.5119

*^a^Each kilogram of mineral premix (0.1% inclusion) supplied the following per kg of complete feed: 60 mg Zn as ZnSO⋅HO; 60 mg Mn as MnSO⋅HO; 40 mg Fe as FeSO⋅HO; 5 mg Cu as CuSO; 1.25 mg I as Ca(IO); and 1 mg Co as CoSO*.

*^b^Each kilogram of vitamin premix (0.1% inclusion) supplied the following per kg of complete feed: vitamin A, 13,200 IU; cholecalciferol, 4000 IU; alpha-tocopherol, 66 IU; niacin, 110 mg; pantothenic acid, 22 mg; riboflavin, 13.2 mg; pyridoxine, 8 mg; menadione, 4 mg; folic acid, 2.2 mg; thiamin, 4 mg; biotin, 0.253 mg; vitamin B, 0.04 mg; ethoxyquin, 100 mg*.

*^c^NaSeO premix provided 0.3 mg Se/kg of complete feed*.

*^d^Feed supplements include XAP, XAP  DFM, and supplemental fat. Approximately 2% supplemental fat was added to create ME difference of 150 kcal/kg. Filler was added in diets that had no feed supplement*.

*As determined by chemical analysis.

#### Bird Husbandry and Tissue Sampling

Eight hundred sixty-four 1-day old female poults (Hybrid Converter Hens, Cold Springs Farm, Thamesford, Ontario, Canada) were randomly assigned to 48 litter floor pens containing 18 poults per pen. Six replicate pens were randomly assigned per dietary treatment. The poults had access to *ad libitum* feed and water and raised according to standard commercial practices. At 42 days of age, six birds per treatments were euthanized by cervical dislocation to collect tissue for histological assessment. A 6-cm section from the mid-portion of the jejunum was taken from each bird, gently flushed with saline, and fixed in 10% formalin solution for subsequent morphometric analysis. Ileal sections were also taken each of the euthanized birds for mucosal adherent mucin secretion as previously described.

### Histological and Histochemical Analysis

#### Histological Analyses

The tissue samples were immediately rinsed with saline, and fixed in 10% neutral-buffered formalin solution for at least 72 h before processing. A total of four sections of about 2–3 mm in length were taken from a 3-cm fixed jejunum section collected from each sampled bird. These smaller sections were placed in tissue cassettes and submerged in 10% buffered formalin solution until processed at the Histopathology Laboratory (NC State University, College of Veterinary Medicine, Raleigh, NC, USA). The fixed jejunum sections were embedded in paraffin wax, and 5 μm thick transverse sections were cut with a microtome. The 5 μm-cut sections were placed on slides and were stained with Lilee Meyer hematoxylin and counter-stained with eosin yellow. A light-microscope (LEICA-DMR light-microscope, Leica Camera AG, Solms, Germany) was used to visualize the transverse sections placed on slides. The images were captured using a Spot-LTCR digital camera (Diagnostic Instruments, Inc., Sterling Heights, MI, USA) and analyzed using Image Tool software (UTHSCSA Image Tool Software, Version 3.0, the University of Texas, San Antonio, TX, USA). Villus height, villus apical width at the tip of the villus, villus basal width at the crypt-villus junction, crypt depth, and muscularis depth were measured on 10 villi per sampled poult. The villi height:crypt depth ratio for each poult was calculated by dividing the average of the 10 villi heights measured per poult by the average of the 10 crypt depths measured on the same poult. The following mathematical formula was used to determine apparent villus surface area {[(villus tip + villus base)/2] × villus height}, according to Iji et al. (11).

#### Histochemical Analyses – Measurement of Adherent Ileal Mucin Layer Thickness

The epithelial-adherent mucin layer thickness was assessed histochemically with Alcian blue stain, based on the affinity of the stain for mucin (12). The thickness of the ileal mucus adherent layer was estimated based on the modification of Parman’s method (13). A 1-cm section of ileal tissue from each sampled bird was removed and placed in 10 g/L Alcian blue dye solution in buffer containing 160 mmol/L sucrose and 50 mmol/L sodium acetate, pH 5.8. After 6 h of incubation, excess dye was extracted with 250 mmol/L sucrose. The absorbed dye was extracted from the tissue by incubation in 10 g/L docusate sodium salt solution overnight at room temperature. Samples were centrifuged at 700 g, plated on 96-well plate, and the optical densities were measured at 620 nm using Alcian blue solution as a standard. The amount of absorbed dye was reported as micrograms of Alcian blue per gram of ileal tissue.

### Statistical Analysis

The experiments were analyzed as completely randomized designs. Pen or cage means were respectively used as the experimental unit for the statistical analysis of adherent ileal mucin secretion. For histological analysis, 10 villi measurements were averaged per tissue sample collected, and this average number served as the experimental unit for the statistical analysis. Data were analyzed using JMP software (Version 10, SAS Institute, Cary, NC, USA). ANOVA was used to examine the main effect of dietary treatment factors, and their interaction on parameters evaluated. Means were separated using the LS Means at *P* < 0.05.

### Animal Ethics

Care of the birds used in all experiments conformed to the Guide for Care and Use of Agricultural Animals in Research and Teaching (14). Moreover, all animal husbandry practices and euthanasia performed during the conduct of these experiments were conducted according to protocol # 12-014-A approved by Institutional Animal Care and Use Committee at North Carolina State University.

## Results

### Experiment 1

Table 3 summarizes the effect of dietary energy level and β-mannanase supplementation on villi morphological characteristics and ileal adherent mucin thickness layer of turkey hens at 21 days of age.

**Table 3:** **Effect of dietary energy level and **β**-mannanase supplementation on jejunum villi morphological characteristics and ileal adherent mucin thickness layer of turkey hens at 28 days of age**.^c^

Main effect		Tip width	Villi height	Base width	Crypt depth	Muscularis thickness	Surface area	Villi height/crypt depth	Ileal adherent mucin thickness layer
		
		Micron					Micron^d^		**μ**g/g tissue
**Energy level**									
High energy		173	1661	301^b^	244	256	393,523^b^	6.19	752
Low energy		207	1768	366^a^	222	266	496,505^a^	6.97	804
**β-Mannanase level (%)**									
0.05		219^a^	1714	367^a^	224	251	509,870^a^	7.49^a^	804^b^
0.00		161^b^	1715	300^b^	242	269	380,157^b^	5.70^b^	832^a^

**Interactions**									

**Energy level**	**β-Mannanase level (%)**								
High energy	0.05	204	1733^a,b^	335	232	266	468,246	6.97^a,b^	631^b^
High energy	0.00	143	1588^b^	266	255	243	318,800	5.43^b^	977^a^
Low energy	0.05	235	1694^a,b^	399	215	236	551,495	7.94^a^	791^a,b^
Low energy	0.00	179	1842^a^	334	228	296	441,514	5.92^a,b^	873^a,b^

**Source of variations**									
					*P*-values			

Energy		0.141	0.097	0.011	0.373	0.586	0.003	0.149	0.1616
β-Mannanase		0.017	0.983	0.009	0.470	0.383	0.001	0.006	0.0466
Energy × β-mannanase		0.909	0.030	0.928	0.831	0.060	0.501	0.025	0.0261
SEM(40)^d^		10	29	11	11	10	14,242	0.35	27.51

*^a,b^Means with different letter superscripts within a column are significantly different (*P* < 0.05)*.

*^c^Values are means of four replicates each treatment*.

*^d^SEM(40), standard error of the mean with 40 degrees of freedom*.

#### Gut Morphological Development

There were no significant treatment effects observed on jejunum mucosa morphology at 7 and 14 days; however, there was a significant treatment effect observed at 28 days. Increasing dietary energy level was associated with an 18% decrease in the villus base width and a 21% decrease in villus surface area (*P* < 0.05). In contrast, dietary supplementation of β-mannanase increased the villus tip width by 36% and villus height/crypt depth by 32% (*P* < 0.05), while the villus base width and surface area was increased by about 22.5 and 34%, respectively (*P* < 0.05).

#### Ileal Adherent Mucin Layer Thickness

There was no significant main effect of dietary energy; however, poults fed the β-mannanase supplemented diet had a 4% reduction in ileal adherent mucin layer thickness as compared to the unsupplemented control diet. Furthermore, the supplementation of β-mannanase to the high-energy diet reduced the ileal adherent mucin layer thickness by about 36% as compared to the high-energy diet without supplementation (*P* < 0.05), but neither of these diets were significantly different from the results observed among birds fed the low-energy diets with or without the enzyme supplementation (*P* > 0.05).

### Experiment 2

Table 4 summarizes the influence of dietary DDGS level, and XAP and DFM supplementation on the morphological measurements of jejunum villi and mucosa of turkey hens at 42 days.

**Table 4:** **Influence of dietary DDGS level, and XAP and DFM supplementation on the morphological measurements of jejunum villi and mucosa of turkey hens at 42 days**.^c^

Main effect		Tip width	Crypt depth	Muscularis thickness	Surface area	Ileal adherent mucin thickness layer
		
		Micron			Micron^d^	**μ**g/g tissue
DDGS level (%)						
6		275^a^	166	251^b^	684,224^a^	679^b^
18		233^b^	169	294^a^	606,656^b^	705^a^

Feed supplements						
Negative control (NC)		264	176^a^	285	670,514	740^a^
~2% Supplemental fat (suppl. fat)		267	180^a^	274	639,138	770^a^
XAP		240	157^c^	266	618,222	589^b^
XAP + DFM		246	159^c^	264	653,887	670^a,b^

**Interactions**						
DDGS level (%)	Feed supplement					
6	NC	296	177	268	735,289	737
6	Suppl. fat	295	185	255	660,544	726
6	XAP	252	145	244	630,957	576
6	XAP + DFM	260	157	238	710,104	679
18	NC	232	176	303	605,737	744
18	Suppl. fat	239	174	293	617,733	814
18	XAP	228	167	288	605,486	603
18	XAP + DFM	233	161	291	597,670	661

Source of variations						
*P*-values						

DDGS		0.002	0.614	<0.01	0.037	0.045
Feed supplement		0.373	0.037	0.334	0.740	0.032
DDGS × feed supplement		0.576	0.423	0.885	0.664	0.871
SEM(40)^d^		12	6.519	13	34,110	46

*^a,b^Means with different letter superscripts within a column are significantly different (*P* < 0.05)*.

*^c^Values are means of six replicates per treatment*.

*^d^SEM(40), standard error of the mean with 40 degrees of freedom*.

#### Gut Morphological Development

Some effects were observed on mucosa morphology development at 42 days of age. Dietary inclusion of 18% DDGS was associated with a 15% decrease in villus tip width (*P* < 0.05) and 11% decrease in villi surface area (*P* < 0.05) relative to the 6% DDGS treatment. However, the 18% dietary inclusion of DDGS treatment had a 13% increase in muscularis thickness (*P* < 0.05) relative to the 6% DDGS inclusion. DDGS inclusion did not have significant effect on the villi height, crypt depth, villi height/crypt depth, and villi base width. The dietary supplements did not have any effect on villi tip width, height, villi height/crypt depth, villi base width, muscularis thickness, and villi surface area, although they did affect the crypt depth. Poults-fed diets supplemented with XAP alone or in combination with DFM had reduced crypt depth by about 12 and 11%, respectively, when compared with the supplemental fat and NC (*P* < 0.05).

#### Ileal Adherent Mucin Layer Thickness

The results of the ileal adherent mucin layer thickness are also presented in Table 4. Increasing the dietary level of DDGS increased the ileal adherent mucin layer thickness observed in poults at 42 days of age (*P* < 0.05). However, in comparison to the supplemental fat and NC treatments, dietary supplementation of XAP reduced the ileal adherent mucin layer thickness by 24 and 20%, respectively (*P* < 0.05); but neither response was different from XAP + DFM.

## Discussion

### Gut Morphological Development

Dietary soluble and insoluble β*-*mannans and arabinoxylans have been reported to exhibit some anti-nutritional properties and adverse effects on growth performance of poultry. The high arabinoxylan content in DDGS along with its inferior amino acid digestibility and variability of other nutrients limits the dietary inclusion of DDGS in poultry feed to <6%. Likewise, the viscous nature of β-mannan in diets, that contain a lot of soybean meal, has been observed to cause physiological and morphological changes to the gastrointestinal tract in poultry, which can impede efficient nutrients utilization (15, 16).

The general hypothesis tested in the two studies reported herein was that the supplementation with β*-*mannanase, XAP, or a combination of XAP + DFM will reduce the anti-nutritional effects and improve apparent nutrient utilization by reducing endogenous nutrient losses associated with enteric mucin secretion, enhancing gut morphological development, and improving gut health. Because dietary inclusion of the enzymes may help degrade the NSPs, their anti-nutritional effects may, thereby, be diminished and thus improve nutrient utilization, as residual enteric substrates are altered to positively favor symbiotic microflora and microbial diversity over the pro-inflammatory pathogenic ones in the hindgut ecosystem, consequently improving the gut health.

Measured changes in intestinal morphology, such as shorter villi and deeper villi crypts, have been used as indicators of gut health and enteric distress (17). Tall mucosal villi increase the surface area available for nutrients absorption (18, 19). There is correlation between the crypt depth and the rate of proliferation of the epithelial cells (20, 21). Epithelial regeneration starts from the villi crypt, so a deep crypt is an indication of rapid enterocyte turnover and increased mucosal tissue maintenance requirements (17, 22). The rapid enterocyte proliferation and the epithelial cell turnover rate greatly impacts protein and energy requirements of the small intestine mucosa (23). Diet composition may produce microscopic alterations in the intestinal mucosa, and it is possible that the change in morphology of the gastrointestinal tract may be associated with dietary NSP levels (24).

Some studies have demonstrated that enzyme treatment can influence the morphological development of intestinal villi (19, 25, 26). In the first trial, we observed that dietary inclusion of β-mannanase had a positive effect on jejunum mucosal morphology among poults sampled at 28 days: villus surface area and tip and base widths were all increased. Although there are few publications demonstrating the effect of β-mannanase on gut morphology, the work of Mehri et al. (27) corroborated our findings. There was an increase in the villi surface area and jejunum villi height/crypt depth ratio by dietary β*-*mannanase supplementation. Mehri et al. (27) observed an increase in villus height and crypt depth in the duodenum when β*-*mannanase was supplemented to corn–soy diets at 700 and 900 g/ton.

In the second experiment, the level of arabinoxylan, a NSP anti-nutritional factor (ANF), was calculated to increase by about 50% as the dietary inclusion level DDGS increased from 6 to 18% (Table 2). Choct and Annison (28) have shown anti-nutritional effects of depressed apparent metabolizable energy when 3% pentosans, primarily comprised of arabinoxylans, was added to broiler diets. Relative to the 6% DDGS treatment, dietary inclusion of 18% DDGS adversely affected jejunum mucosal morphology as indicated by reduced villi surface area, tip width and increased thickness of the villi muscularis. The poor jejunum morphological development may be associated with relatively higher NSP content in the 18% DDGS diet. Iji (29) reported that NSP can negatively impact gut morphology by increasing crypt depth of both the jejunum and ileum, thus accelerating enterocyte turnover. Although we evaluated the effects of a blend of supplemental enzymes containing xylanase in the second experiment, xylanase supplementation alone has been reported to increase villus height of the duodenum, jejunum, and ileum, and the villus height/crypt depth of these three segments (19). Furthermore, the dietary inclusion of DFM may also impact the gut morphology by increasing the jejunum and ileal villi heights (30, 31). Dietary supplementation of the enzyme blend of XAP and XAP + DFM combination had a beneficial effect on gut morphology development. The addition of the enzyme blend with or without the DFM reduced the crypt depth, indicating reduced mucosal distress, in comparison to the NC or fat-supplemented diet.

Apparently, the effects observed on enteric mucosa morphology in both trials may have been associated with the changes in the substrate characteristics within the enteric ecosystem by the dietary supplementations (β*-*mannanase, XAP, and XAP + DFM), which in turn altered the fermentation of resident enteric microflora (32).

### Adherent Ileal Mucin Secretion

Establishment of a symbiotic enteric ecosystem minimizes the inflammatory symptoms of enteric distress caused by the proliferation of enteric pathogens, which is associated with increased intestinal mucin secretion (33, 34). Parsaie et al. (35) reported that distressed intestinal morphology, as indicated by the shortened villi and deepened crypts, may cause increased mucosal secretions, primarily as mucin. Indeed, some research reports have shown that the dietary inclusion of enzymes reduce the mucosal blanket secretions in monogastric animals (36, 37).

In addition to the improved gut morphological development observed in this study, a reduction in the ileal adherent mucin layer thickness was observed with dietary β-mannanase supplementation, especially in the high-energy (fat) diets. Few studies have been reported on the influences of β*-*mannanase on mucin secretion. However, research results reported trial by Mehri et al. (27) indirectly validated our observation. They reported that dietary β*-*mannanase supplementation reduced the number of mucosal goblet cells per unit of epithelial surface area in broilers. Since mucin is secreted by the epithelial goblet cells, this observation agrees with our observations that dietary β*-*mannanase supplementation significantly reduces intestinal adherent mucin layer thickness.

We also observed that the addition of XAP reduced ileal mucin thickness layer. Bedford et al. (36) also noted that xylanase supplementation improved the apparent digestibility of threonine, which was associated with a decrease in intestinal mucin secretion. Pirgozliev et al. (37) also observed a decrease in the quantity of threonine excreted by the birds fed with a xylanase-supplemented diet, which he attributed to less secretion of intestinal mucin.

## Conclusion

Improved gut morphological development of the mucosa villi in the jejunum and ileum and the reduced adherent ileal mucin thickness layer could likely contribute to the improved apparent nutrient utilization, which reflects in the better growth performance in poultry often observed when their diets are supplemented with exogenous enzymes. Enteric microflora profile has been demonstrated to be influenced by nutrient abundance (32). Digesta viscosity increases as dietary anti-nutritional NSPs increases, which impairs foregut digestion and absorption of fats, starches, and proteins, and causes more of these nutrients to pass to the hindgut where they “feed” competitive pathogenic bacteria. Proliferation of pathogenic bacteria, like *C. perfringens*, causes enteric tissue inflammation, mucosal leakage, and increased mucin secretion as a protective innate immune response. With protein being a major component of mucin, this increase in mucin secretion can further exacerbate the proliferation of putrefying bacterial pathogens and also contribute to the significantly reduced apparent nitrogen retention. Dietary supplementation of NSP enzymes can be an effective means to counter the adverse effects of high digesta viscosity and cause the hind gut microflora to shift toward a more symbiotic ecosystem.

The research results reported herein demonstrate that dietary supplementation of either β*-*mannanase, XAP, or XAP + DFM can improve gut health in turkey poults as indicated by improved morphological development of the enteric mucosa and reduction in adherent ileal mucin secretion, thereby increasing productive use of nutrients toward better growth performance. Further studies are still needed to evaluate the effect of other supplemental enzymes on gut health, especially as the trend of limiting the use of antibiotic growth promoters in animal feed continues.

## Conflict of Interest Statement

The authors declare that the research was conducted in the absence of any commercial or financial relationships that could be construed as a potential conflict of interest.
